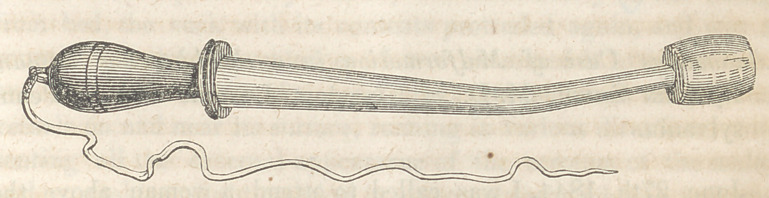# Galvanism and Electro-Magnetism, in the Treatment of Uterine Hæmorrhage, Prolapsus Uteri, &c.

**Published:** 1848-04

**Authors:** Tracy E. Waller


*Galvanism and Electro-Magnetism, in the Treatment of Uterine Hæmorrhage, Prolapsus Uteri, &c.* By Tracy E. Waller, M. D.

In consideration of the great power of galvanism as a curative agent, and the fact that its use is yet very limited, I am induced to offer a few remarks on its application in the treatment of certain uterine affections. I believe Dr. Radford, of Manchester, England, was the first to apply it in uterine hæmorrhage. He used it in inertia of the womb, during labour and after delivery, with the most gratifying results. Thomas Dorrington, Esq., Surgeon to the Manchester and Salford Lying-in Hospital, applied it in a case of placenta prævia, and in one or two other cases of hæmorrhage in the latter months of pregnancy. Henry Wilson, Esq., Surgeon, Runcorn, and Mr. Clarke, of Dublin, have likewise used it successfully in similar cases, and perhaps others on that side of the Atlantic. The instrument used for a conductor by Mr. Wilson, was made of copper wire wound with thread and covered with sealing wax, with a small ball of sponge fastened to the end to be applied to the womb. The one used by Dr. Radford, was of copper wire coated with sealing wax varnish, with a silver ball attached to one end by a screw, and connected with the machine by a wire and spiral spring concealed in the handle. Mr. J. P. Stratton of this city has recently contrived an instrument which appears to me to be better adapted and more conveniently applied than either of these.

This is made of seasoned wood, of proper size and length, and bent to suit the vaginal curve in the direction of the womb: through this a copper wire passes, with a metallic ball one inch in its longest and three-fourths of an inch in its shortest diameter, fastened by a screw to the end and coated with silver. The wire from the machine is attached at the handle end of the instrument by means of a small hole through the conducting wire or rod. The handle is turned, and of convenient size. The wood from the ball to the handle is well coated with sealing wax varnish, and it is thus rendered a very neat and durable instrument. When applied to the womb, I have observed that contraction immediately follows. The manner of making the application is as follows: A piece of flannel, wet in alcohol or spirits, should be laid over the abdomen of the patient; on this the positive pole is to be held by an assistant, while the physician applies the instrument attached to the negative pole introduced into the vagina, and resting on the os uteri. Dr. Golding Bird says, that "galvanism is probably the very best emmenagogue we have." It may be employed, no doubt, with success and perfect safety in all passive forms of uterine hæmorrhage; and in that common and most distressing complaint, prolapsus uteri, I have reason to believe it will be a highly useful remedy. I have never seen any account of its employment in this disease, but having used it myself in a few cases, I can recommend it as a most valuable agent. It should be remembered that the negative pole of the battery is attached to the uterine conductor, and the positive applied to the abdomen just above the pubis. I have used galvanism in dysmenorrhœa also with the most gratifying results: and in a variety of other complaints I have found it to be a remedy of great value, an account of which I hope ere long to present to the profession.

## Figures and Tables

**Figure f1:**